# Intraoperative hepatic subcapsular spider-like telangiectasia sign for the definitive diagnosis of biliary atresia

**DOI:** 10.1186/s12887-022-03831-z

**Published:** 2023-02-06

**Authors:** Kaizhi Zhang, Yan Tang, Rui Liu, Zebing Zheng, Chengyan Tang, Yuanmei Liu, Zhu Jin

**Affiliations:** 1 Department of Pediatric Surgery, Affiliated Hospital of Zunyi Medical University, Zunyi, 563000 China; 2 Department of Pediatric Surgery, Guizhou Children’s Hospital, Zunyi, 563000 China; 3 Clinical Medical College, Chengdu University, Chengdu, 610081 China

**Keywords:** Biliary atresia, Hepatic subcapsular spider-like telangiectasia sign, Ultrasonic examination, Liver stiffness value, Diagnosis

## Abstract

**Objective:**

To evaluate the accuracy of intraoperative hepatic subcapsular spider-like telangiectasia (HSST) sign for differentiating biliary atresia (BA) from other causes of hepatic cholestasis.

**Methods:**

The data of 69 patients with jaundice treated from January 2019 to December 2021 were retrospectively analyzed. Based on intraoperative cholangiography (IOC), the patients were divided into two groups: the BA group (*n* = 49) and the non-BA group (*n* = 20). The biochemistry tests, liver ultrasound, liver stiffness value and HSST sign of the two groups were compared.

**Results:**

The incidence of abnormal gallbladder, elevated γ-glutamyl transpeptidase (γ-GGT) > 182.0U/L and abnormal liver stiffness (> 6.4 kPa) in BA group were significantly higher than those in non-BA group (*P* < 0.001). The HSST sign was present in all BA patients and not found in non-BA group. The area under receiver operating curve of direct bilirubin(DBIL), γ-GGT, abnormal gallbladder, liver stiffness value and HSST sign were 0.53, 0.84, 0.78, 0.96, and 1.00, respectively. The sensitivity, specificity, positive predictive value (PPV) and negative predictive value(NPV) of HSST sign in the diagnosis of BA were all 100%.

**Conclusion:**

Presence of HSST sign on diagnostic laparoscopy is highly suggestive of BA.It can be used in the differential diagnosis of BA and non-BA.

**Level of evidence:**

Level III.

## Introduction

Biliary atresia (BA) is characterized by persistent jaundice, acholic stools and progressive liver fibrosis. Its etiology is unclear [1–3]. If not diagnosed and treated in time, BA can rapidly progress to liver cirrhosis and the child usually dies at around 2 years old [4–6]. Currently, Kasai portoenterostomy is the treatment of choice for BA, which can effectively restore bile drainage, delay the development of liver cirrhosis and improve the survival rate [7]. Therefore, the early diagnosis of the disease is particularly important to improve the prognosis. Currently, liver ultrasound, liver stiffness value and biochemical tests are used for the early diagnosis of BA. However, the accuracy and specificity of these tests are variable [8–11]. The definitive diagnosis of BA is made by intraoperative cholangiography (IOC) and liver biopsy [12–14]. However, due to the invasiveness of the procedure and the need for radiation, IOC is not readily accepted by the patients' families leading to a delay in the diagnosis. Hence, timely differentiation of BA from other causes of cholestatic diseases in children remains a challenge [15].

Previous studies have reported that most of the BA patients have abnormal blood flow signals under the liver capsule on color Doppler ultrasonography [16, 17]. During Kasai’s operation, multiple subcapsular spider-like telangiectasis lesions called as HSST sign are visible in BA patients. Zhou et al. first reported that HSST sign was used as a marker for diagnosing BA [18]. In this study, we evaluated the HSST sign using diagnostic laparoscopy in BA patients and those with other causes of cholestatic jaundice. We also compared their biochemical tests, liver ultrasound findings, liver stiffness value and IOC.

## Materials and methods

### Ethical approval of the study protocol

The study protocol was approved by the Affiliated Hospital of Zunyi Medical University.

### Data collection

The clinicoradiological data of 103 children with BA and other causes of cholestasis admitted to the Department of Pediatric surgery of the affiliated Hospital of Zunyi Medical University from January 2019 to December 2021 were collected and retrospectively analyzed. A total of 34 were excluded. Children were excluded if 1) preoperative or intraoperative data was missing, 2) IOC was not performed in our hospital, and 3) children who could be definitive diagnosed by other preoperative examinations.

The demographic data (age, sex,weight), total bilirubin (TBIL), direct bilirubin (DBIL), γ-gamma-glutamyl transferase (γ-GGT), alkaline phosphatase (ALP), alanine aminotransferase (ALT), aspartate aminotransferase (AST), liver ultrasound, liver stiffness value and HSST sign were recorded and analyzed.

**Fig. 1:**
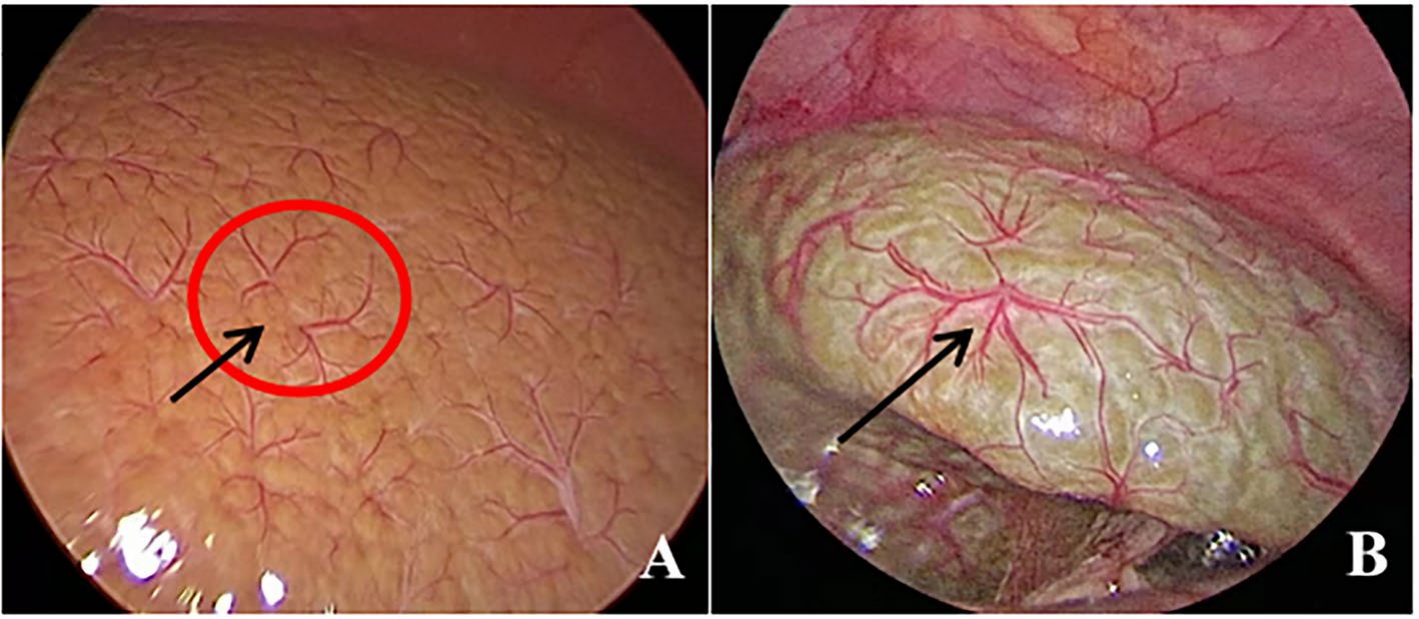
Two types of HSST sign in BA. **A** Decentralized type of HSST sign in a 69-day-old boy with BA (black arrow). **B** Centralized type of HSST sign in a 75-day-old boy with BA (black arrow)

All patients underwent diagnostic laparoscopy and laparoscopic IOC under general anesthesia. The pneumoperitoneum needle was placed 3 mm from the upper left edge of the umbilical ring, filled with CO_2_, and the pneumoperitoneum pressure was maintained at 6 mmHg. The 3 mm trocar was placed, and the texture, color, appearance, HSST sign and gallbladder of the liver were detected with a video camera. The HSST sign was defined as the presence of three or more branches in a large tortuous spiderlike vascular plexuses distributed on the liver surface in a decentralized (radial branches arising from more than one central point, but close to each other to form a spiderlike sign) (Fig. 1A) or concentrated (radial branches arising from a central point) pattern (Fig. 1B) [18].

The indications of IOC were long duration of jaundice (> 2 weeks) with depigmented stools, DBIL > 20%TBIL, and inability to make definitive diagnosis of BA preoperatively [19]. A 3 mm trocar was placed at the right edge of the umbilical ring as the operating hole, and the projection point of the gallbladder on the right upper abdomen was cut by 3 mm to raise the gallbladder for IOC.The gallbladder was examined for wall thickness (or residues) and intubated with thin needles. Then a water-soluble contrast was injected with high pressure through the needles in to the gallbladder and X-ray of the upper abdomen was taken. Free passage of the contrast agent in to the duodenum and intrahepatic bile ducts ruled out the diagnosis of BA. Based on the IOC findings, children are divided into BA group and non-BA group.

Abnormal gallbladder was defined as stiff, small, irregular, or absent gallbladder on abdominal ultrasound [8, 20]. The liver stiffness value was measured by abdominal ultrasound shear wave elastography. The ultrasound and shear wave elastography examinations were performed by the radiologists with at least 5 years’ experience on a LOGIQ E9 scanner (GE Medical Systems, the United States). All scans were carried out with a ML6-15 linear array probe (5-13 MHz). The right intercostal space of the child was selected uniformly. With the aid of two-dimensional ultrasound, the thickness of liver parenchyma was selected, which met the probe requirements and had no big blood vessels. Five different sections of the liver were selected multiple times for the measurement and the median value was taken as the final result by the image data processing system. The liver stiffness value was expressed in kPa. The normal range of liver stiffness values was less than 4.6kpa. The biochemical tests were conducted within 7 days before operation and after 8 h of fasting. Laparoscopic liver surface images were blindly evaluated by two observers for the presence of HSST sign.

### Statistical analyses

Using SPSS 26.0 software, continuous variables were expressed as medians and interquartile ranges (IQR),Qualitative data were represented by counts. Continuous variables were analyzed by using the non-parametric test and the qualitative data was compared by chi-square test or Fisher’s exact test. Receiver operating characteristic (ROC) curve analysis determined the optimal critical value of diagnostic methods. Sensitivity, specificity, positive predictive value (PPV), and negative predictive value (NPV) were used to determine the accuracy of HSST sign and other diagnostic methods for BA.

## Results

Based on the selection criteria, 34 children were excluded and 69 children were enrolled in this study. There were 43 boys and 26 girls. Diagnoses of BA patients were all type III BA (*n* = 49). The diagnoses of patients in non-BA group were cytomegalovirus hepatitis (*n* = 6), idiopathic neonatal hepatitis (*n* = 10), progressive familial intrahepatic cholestasis (*n* = 3), and Alagille syndrome (*n* = 1).

There were 32 boys (65.3%) in the BA group and 11 boys (55.0%) in the non-BA group (*P* = 0.429). At the time of admission, the median age was 63 days (IQR,50–92) and 56 days (IQR,29.5–68.75) in the BA and non-BA groups, respectively (*P* = 0.472). The median age at the time of the operation was 67 days (IQR,55–94) and 60.5 days (IQR,37.25–74.5) in the BA and non-BA groups, respectively (*P* = 0.572) (Table 1).

**Table 1:** Comparison of patient characteristics between the BA and non-BA groups

Parameters	BA (*n* = 49)	Non-BA (*n* = 20)	*P*-value
Sex (female/male)	17/32	9/11	0.429
Age at the time of admission (days)	63 (50–92)	56 (29.5–68.75)	0.472
Age at the time of operation (days)	67(55–94)	60.5 (37.25–74.5)	0.572
Weight(kg)	5.3 (4.5–6.5)	4.65 (3.4–5.1)	0.045
TBIL (μmol/L)	178.4(152.5–203.6)	170.6 (147.4–298.5)	0.851
DBIL (μmol/L)	95.6 (83.7–108.9)	93.8 (70.6–126.8)	0.851
ALT (U/L)	152 (113–234)	110.5 (82–182)	0.211
AST (U/L)	266 (206–369)	199.5 (108.9–209.3)	0.273
GGT (U/L)	331 (185–736)	109 (85.3–160)	0.001
ALP (U/L)	751 (550–969)	520.5 (426–766.5)	0.211
Liver stiffness value (kPa)	10.7 (8.4–13.4)	4.8 (3.8–5.2)	< 0.001
Abnormal gallbladder	47/49 (95.9%)	8/20 (40.0%)	< 0.001
HSST sign	49/49	0/20	< 0.001

**Fig. 2:**
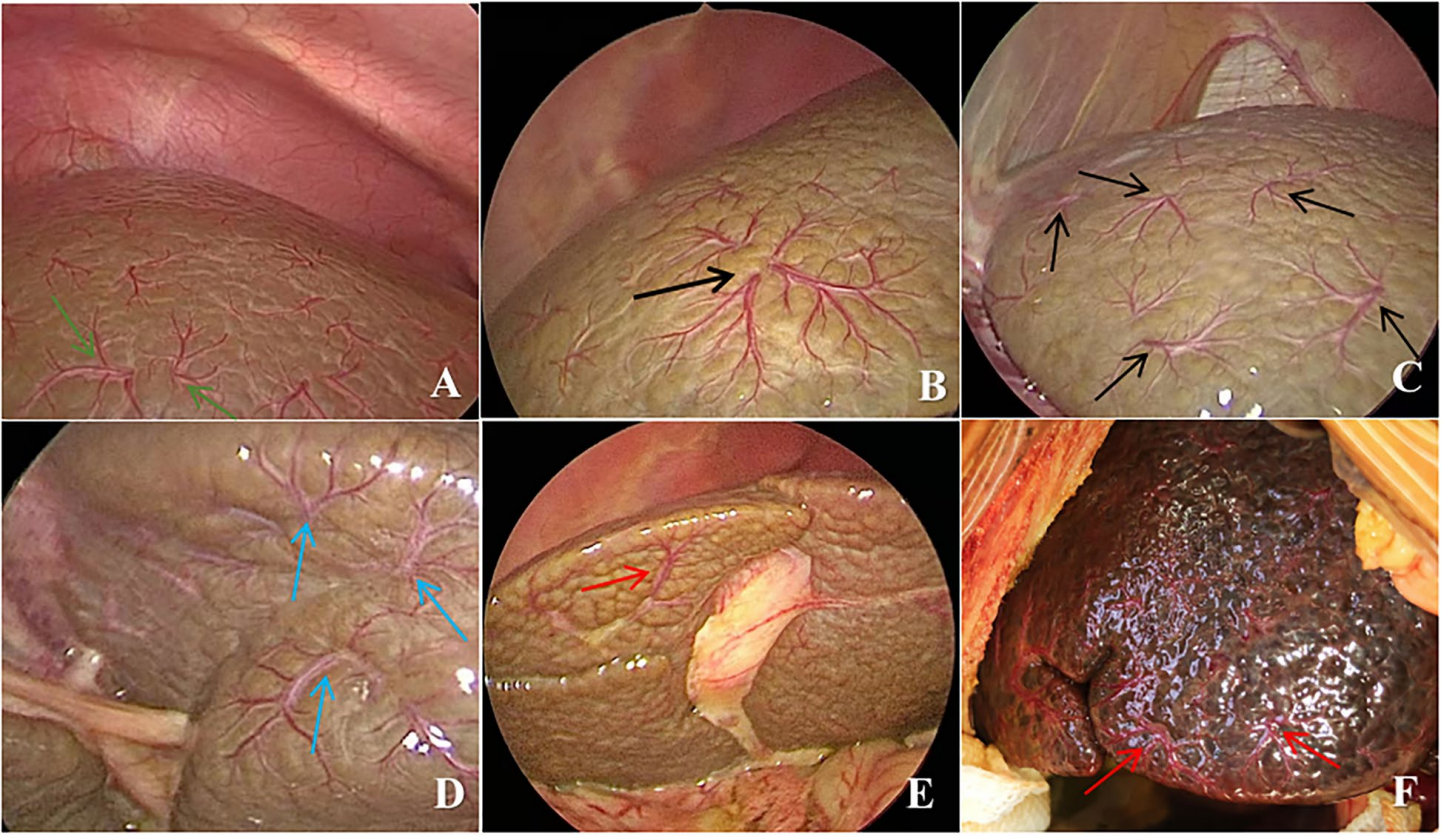
Laparoscopic view of the liver surface in BA patients. **A** HSST sign in a 79-day-old boy with BA (green arrows). **B** HSST sign in a 72-day-old girl with BA (black arrow). **C** HSST sign in a 83-day-old girl with BA (diaphragmatic surface, black arrows). **D** HSST sign in a 83-day-old girl with BA (visceral surface, blue arrows). **E** HSST sign in a 90-day-old boy with BA (red arrow). The gallbladder was small and the liver surface was granular and nodular. **F** HSST sign was seen at open surgery in a 66-day-old boy with BA (red arrows)

**Fig. 3:**
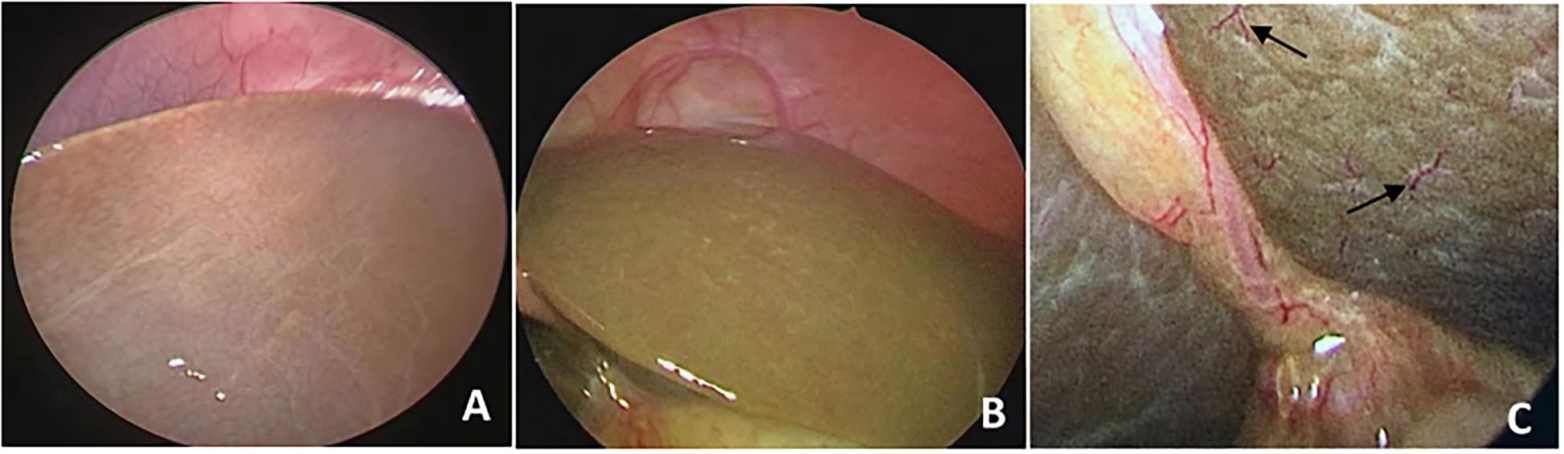
Laparoscopic view of the liver surface in non-BA patients. **A** The liver surface of a 58-day-old boy with idiopathic neonatal hepatitis (visceral surface). The liver surface was smooth and no HSST sign. **B** The liver surface of a 63-day-old girl with cytomegalovirus hepatitis (diaphragmatic surface). The infant had severe cholestasis and no HSST sign. **C** The liver surface of a 74-day-old boy with progressive familial intrahepatic cholestasis. A small number of vascular hyperplasia (black arrows) can be seen on the liver surface, with short branches

There was no significant difference in TBIL level, DBIL level, ALT level, AST level, and ALP level between the BA group and non-BA group (*P* = 0.851, *P* = 0.851, *P* = 0.211, *P* = 0.273, and *P* = 0.211, respectively), while weight, and GGT level were statistically different between the two groups (*P* = 0.045, and *P* = 0.001, respectively). The incidence of abnormal gallbladder and liver stiffness values in the BA group were significantly higher than those in the non-BA group (*P* < 0.001) (Table 1). Eight non-BA patients had abnormal gallbladder, of which six cases had small gallbladder and thick wall, which were diagnosed as progressive familial intrahepatic cholestasis (*n* = 1), cytomegalovirus hepatitis (*n* = 1) and idiopathic neonatal hepatitis (*n* = 4); two cases with irregular gallbladder were diagnosed as Alagille syndrome (*n* = 1) and idiopathic neonatal hepatitis (*n* = 1), respectively. The HSST sign was easily visible in all BA patients on diagnostic laparoscopy (Fig. 2). The minimum age at the time of operation of the BA patients was 14 days old, and the maximum age was 184 days old. The HSST sign was absent in all patients in the non-BA group, as confirmed by both observers (Fig. 3).

**Fig. 4:**
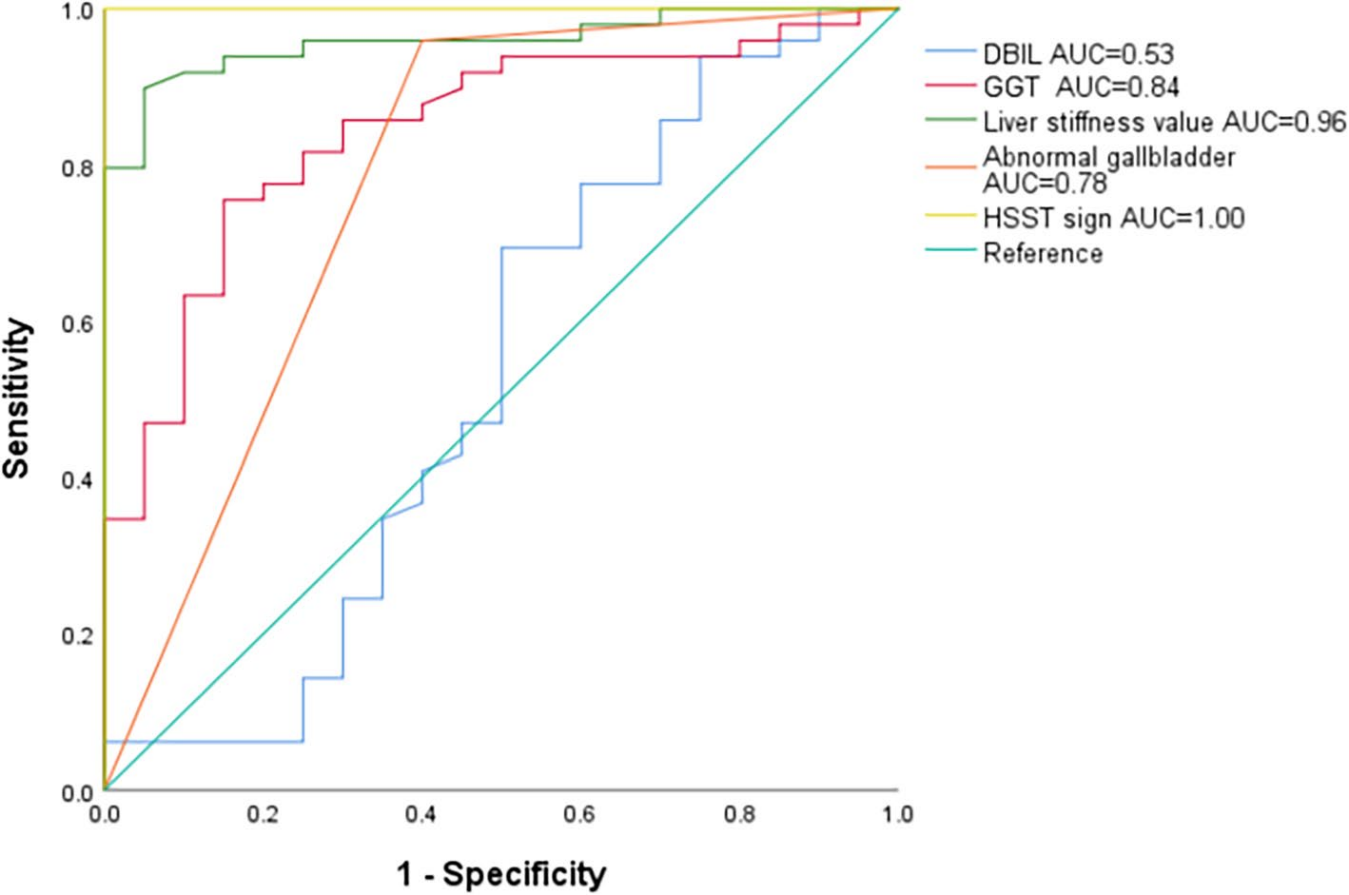
The ROC curves of DBIL, γ-GGT, abnormal gallbladder, liver stiffness value and HSST sign in differentiating BA

The ROC curves of DBIL, γ-GGT, abnormal gallbladder, liver stiffness value and HSST sign showed that area under the curve (AUC) was 0.53, 0.84, 0.78, 0.96 and 1, respectively for the diagnosis of BA (Fig. 4). HSST sign had the highest AUC for diagnosing BA. At the same time, the sensitivity, specificity, PPV and NPV of each index were compared according to the cutoff value (Table 2).

**Table 2:** Differences in the diagnostic accuracy of various parameters

Characteristics	AUC	Sensitivity	Specificity	Positive predictive value	Negative predictive value	Cutoff
DBIL (μmol/L)	0.53	69.4%	50.0%	77.0%	40.0%	87.9
GGT (U/L)	0.84	75.5%	85.0%	92.5%	58.6%	182.0
Liver stiffness value (kPa)	0.96	89.8%	95.0%	97.8%	79.2%	6.4
Abnormal gallbladder	0.78	95.9%	60.0%	85.5%	85.7%	-
HSST sign	1.00	100%	100%	100%	100%	-

## Discussion

BA is one of the important causes of jaundice in infants [21]. The clinical presentation of BA is similar to other cholestatic diseases such as cytomegalovirus hepatitis, idiopathic neonatal hepatitis, progressive familial intrahepatic cholestasis, and Alagille syndrome. Early and timely surgical treatment of BA helps in reducing jaundice, delay liver fibrosis and prolong the survival native liver in children [22]. Therefore, early diagnosis of BA is important. Preoperative diagnosis of BA can be made by various methods such as liver ultrasound, biochemical tests, estimation of liver stiffness, magnetic resonance imaging (MRI), magnetic resonance cholangiopancreatography (MRCP), duodenal tube test, IOC and liver biopsy [8]. For the diagnosis of BA, liver biopsy has a high diagnostic value for BA, but not as high IOC. Hence, IOC is still needed for definite diagnosis.Aleksandra et al. [23] study showed that the duodenal tube test had a high accuracy, with the sensitivity of 97% and specificity of 72%. However, the procedure is complex and has its own limitations. At our center, preoperative diagnosis of BA is done mostly by biochemical tests, liver ultrasound and estimation of liver stiffness. Laparoscopic IOC is performed during surgery to confirm the diagnosis of BA before performing Kasai’s operation. Recently, we noticed that almost all children with BA had HSST sign on laparoscopic exploration of the liver surface as described before. Therefore, we conducted this study to evaluate the accuracy of HSST sign in the diagnosis of BA and compared it with preoperative tests.

In this study, we found that the sensitivity, specificity, PPV and NPV of γ-GGT > 182U/L was 75.5%, 85.0%, 92.5% and 58.6%, respectively. The study by Chen et al. had found γ-GGT > 303U/L to be helpful for the diagnosis of BA before 120 days [19]. Mehmet et al*.* [24] found that γ-GGT > 197U/L to have the sensitivity and specificity of 65% and 79.4%, respectively. Another study by El-guindi et al. [15] suggested that the sensitivity and specificity of γ-GGT > 286U/L was 76.7% and 80%, respectively. Due to the significant differences in the γ-GGT cutoff value and low sensitivity, it cannot be used for definitive diagnosis of BA but it surely helps in suspecting BA in newborns.

**Fig. 5:**
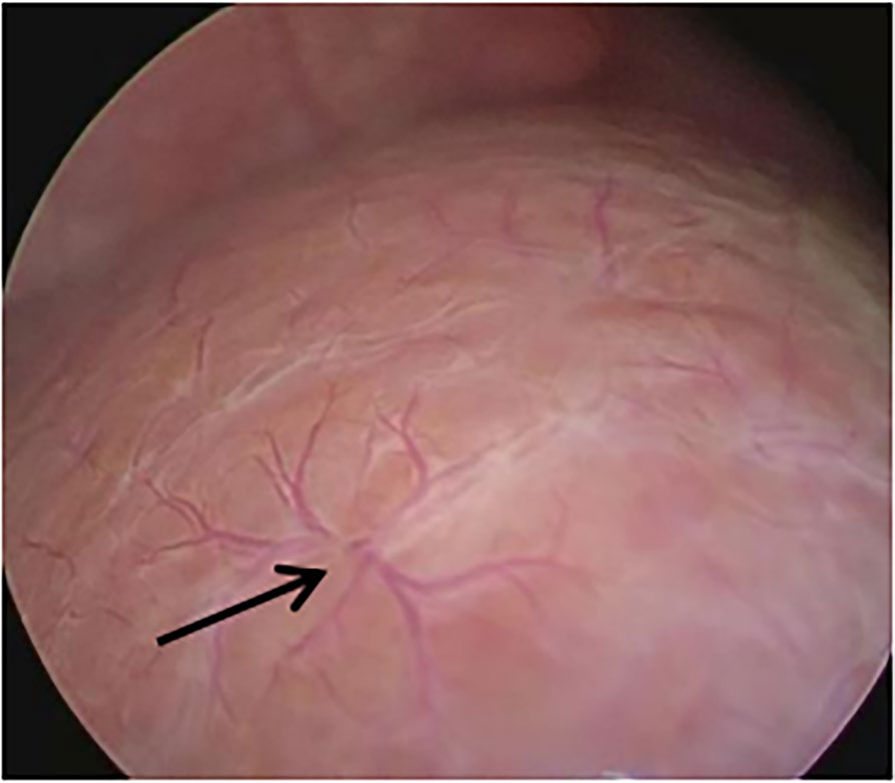
HSST sign in a 14-day-old boy with BA. HSST sign was clearly seen on the liver surface and there was no liver fibrosis (black arrow)

Liver ultrasound is widely used for the diagnosis of BA with the most common ultrasonic signs being the presence of abnormal gallbladder and triangular cord sign [25, 26]. In this study, we found that abnormal gallbladder was present in most of the BA patients but also in 40.0% of non-BA children. In addition, the echo of hepatic hilum was not clear in most children due to which the triangular cord sign could not be appreciated in most of the children with BA. Previous study had reported that the AUC of abnormal gallbladder to be 0.940, the sensitivity of 96.1%, and the specificity of 92.0% [27]. However, in the current study the sensitivity was 95.9%, and the specificity was only 60.0%. In recent years, many researchers and clinicians have used liver stiffness for the early diagnosis of BA [10, 27, 28]. The threshold value of liver stiffness used in our hospital to distinguish BA from non-BA is 4.6kpa. Majority of children with BA in our hospital have liver stiffness value in the range of 8 to 12kpa. In this study we found that the AUC of liver stiffness value > 6.4kpa for diagnosing BA was 0.96, and the sensitivity and specificity were 89.8% and 95.0%, respectively. These findings indicate that liver stiffness is better than γ-GGT and ultrasound for the diagnosis of BA. We found that only two infants with BA had liver stiffness value < 4.6kpa. Both these infants were less than 20-day-old and had no obvious fibrosis in the liver due to which the liver stiffness value was low. However, both of them had obvious HSST sign on the liver surface (Fig. 5).

**Fig. 6:**
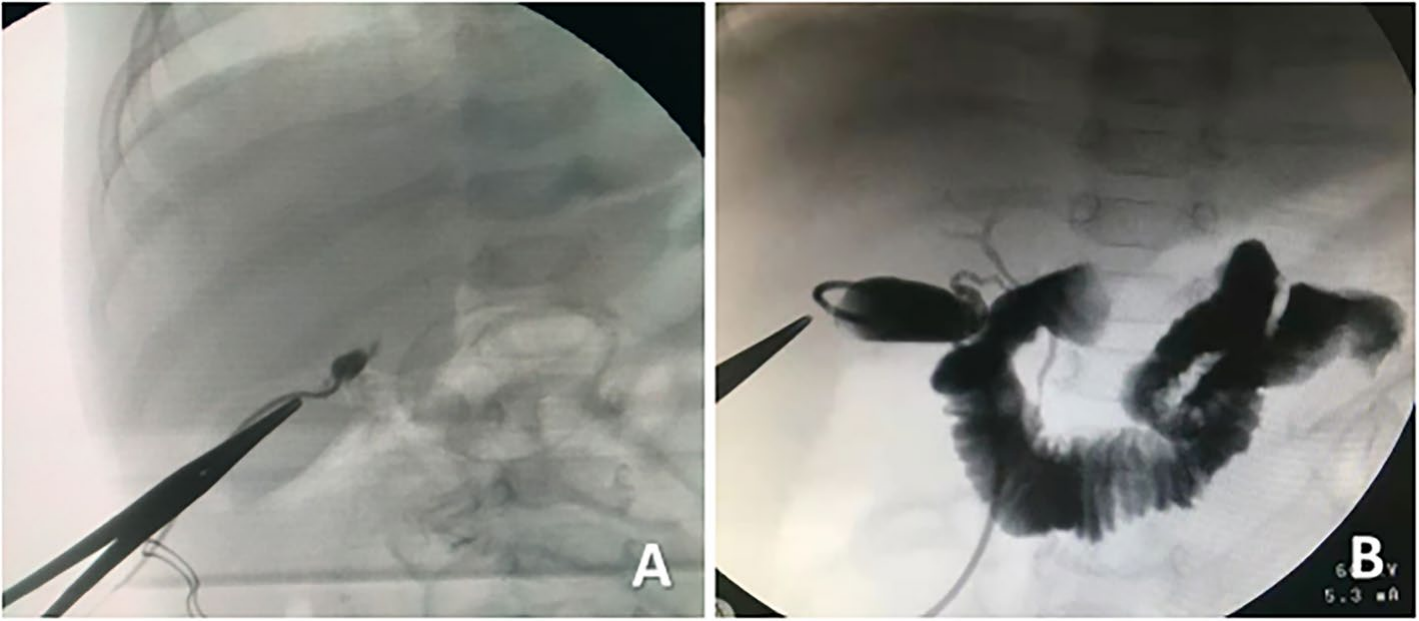
Cholangiography in BA and non-BA patients. **A** IOC in a 61-day-old girl with BA. Both intrahepatic and extrahepatic bile ducts were not visualized. **B** IOC in a 58-day-old boy with cytomegalovirus hepatitis. Both intrahepatic and extrahepatic bile ducts and gallbladder were visualized

IOC is the gold standard for the diagnosis of BA. Currently, laparoscopic IOC is more commonly done (Fig. 6), which usually requires only two to three 3 mm or 5 mm trocars. However, it is an invasive operation and has its own complications. In order to reduce the risk of complications and diagnose BA efficiently, the HSST sign can be used as it has a good diagnostic value with the sensitivity of 100% and specificity of 97.8% [18]. In addition, Li et al*.* [29] reported that the accuracy of laparoscopic HSST sign in the diagnosis of BA is 98.7%, and the laparoscopic HSST sign is superior to cholangiography, which can be used as a highly accurate method to diagnose BA and similar to the results of our study. Therefore, laparoscopic HSST sign is of great significance for the diagnosis of BA.

The etiology of vascular dilatation and hyperplasia on the liver surface of children with BA is unclear. We initially thought that HSST sign might be related to liver fibrosis, but later observed that young patients (younger than 20 days) without liver fibrosis also have HSST sign. Meanwhile, Zhou et al*.* [18] found that 14 newborns aged less than 40 days had HSST sign despite no liver cirrhosis suggesting that HSST sign was not associated with liver cirrhosis. Dilated blood vessels on the liver surface of children with BA are branches of hepatic arteries [30]. After analyzing, we think that most important point of the occurrence of HSST signs may be related to portal hypertension and hypertrophic and hyperplastic changes of hepatic artery branches. Second, the occurrence of multiple branching vessels in the liver of children with BA may be caused by the microenvironment of the disease, which promotes angiogenesis [31, 32]. In the next study, we intend to investigate the correlation between portal vein diameter and portal vein flow, and whether the cause of hepatic artery branch expansion is related to vascular remodeling or abnormal resistance.

The surgical videos of our patients were reviewed by two senior experts and focused on the dilation and proliferation of blood vessels on the liver surface. They found that children with BA had three or more branches of dilated blood vessels on the liver surface and the results correlated with IOC. HSST sign was seen in 49 cases of BA, which was widely distributed on the surface of the liver (Fig. 2A,B). The HSST sign was slightly more on the diaphragmatic surface of the liver than on the visceral surface (Fig. 2C,D). There were 3–8 unequal vascular branches at each site. Additionally, the small gallbladder and granular surface of the liver in some children with BA could be clearly seen on diagnostic laparoscopy (Fig. 2E). The HSST sign could also be clearly seen during open surgery (Fig. 2F). Among the 20 cases of non-BA, 13 cases had no angiogenesis on the liver surface, and the liver surface was smooth, dark red or brown (Fig. 3A,B). In 7 cases, HSST like lesions were seen on the liver surface, with short branches (Fig. 3C). Laparoscopic HSST sign has several advantages. First, laparoscopy is common in almost all hospitals, and simple laparoscopy can make a diagnosis. Second, it is easier to perform and can be done faster than IOC which shortens the operation time [18, 29]. Third, diagnostic laparoscopy to detect HSST sign is a minimally invasive procedure and does not cause radiation exposure to children unlike IOC.

There are some limitations of this study. First, it was a retrospective study with limited sample size. Second, it was a single center study. In the future, we will conduct a multi-center, prospective randomized study to determine the diagnostic ability of HSST sign. Additionally, diagnostic laparoscopy is a minimally invasive procedure, not a non-invasive method for diagnosing BA.

## Conclusion

In summary, the detection of HSST sign by laparoscopy has high accuracy for the diagnosis of BA. The HSST sign is simple, intuitive and high accuracy, it can be a new method to diagnose BA.

## Data Availability

All data generated or analyzed during this study are included in this published article. Data are however available from the corresponding author upon reasonable request and with permission of the interviewees.

## Abbreviations

ALT: Alanine aminotransferase
AST: Aspartate aminotransferase
ALP: Alkaline phosphatase
AUC: The area under the curve
BA: Biliary atresia
DBIL: Direct bilirubin
γ-GGT: γ-Gamma-glutamyltransferase
HSST: Hepatic subcapsular spider-like telangiectasia
IOC: Intraoperative cholangiography
IQR: Interquartile ranges
MRCP: Magnetic Resonance Cholangiopancreatography
MRI: Magnetic Resonance Imaging
NPV: Negative predictive value
PPV: Positive predictive value
ROC: Receiver operating characteristic curves
TBIL: Total bilirubin
